# No More Ovariotomy

**Published:** 1876-06

**Authors:** 


"No More Ovariotomy."—Under the above startling title, says the London *Medical Times and Gazette*, we find a note in the *Surgical Centralblatt* for February 12th, taken from the *Wiener Med. Presse*, 1875, No. 52, by Dr. Semeleder. About two years ago he was informed that a lady of his acquaintance, suffering from an ovarian cyst, who had been much relieved by acupuncture (? galvanopuncture) had been ultimately cured in Vienna by the same treatment. Since that time he has tried it in three cases:

1. A young lady, aged eighteen, who had a soft, fluctuating tumor, originating on the left side, and extending three centimeters above the umbilicus, was subjected to galvanopuncture. In four months the diameter of the abdomen, two inches below the umbilicus, was reduced from ninety-six centimeters to ninety-two centimeters, and in two months more the cure was completed.

2. A lady twenty-four years old, and the mother of two children, had a tumor in the lower part of the abdomen, on the left side, as large as the head of a child two years old. When she had been under treatment two months the patient was cured, the remains of the cyst being hard, and of the size of a small apple.

3. A woman forty years of age, with a tumor reaching up to the umbilicus, had so far recovered at the end of six weeks of the treatment that its continuance was considered unnecessary.

No unpleasant consequences occurred in these cases, and none of the cysts have refilled. The author considers that the action is the same as that which occurs when the poles of a battery are placed in an albuminous fluid, viz., clotting and thickening at the positive pole, and liquefaction at the negative. He considers the method equally applicable to multilocular and unilocular cysts. He does not give an exact account of his method of procedure, but each sitting was of short duration. He anticipates equally favorable results in the treatment of hydatid cysts on this plan.

